# Supplementary figures and images for: Pseudomonas aeruginosa type IV pili actively induce mucus contraction to form biofilms in tissue-engineered human airways
Source: PLoS Biol. 2023 Aug 1;21(8):e3002209. doi: 10.1371/journal.pbio.3002209 (PMC10393179; doi:10.1371/journal.pbio.3002209)

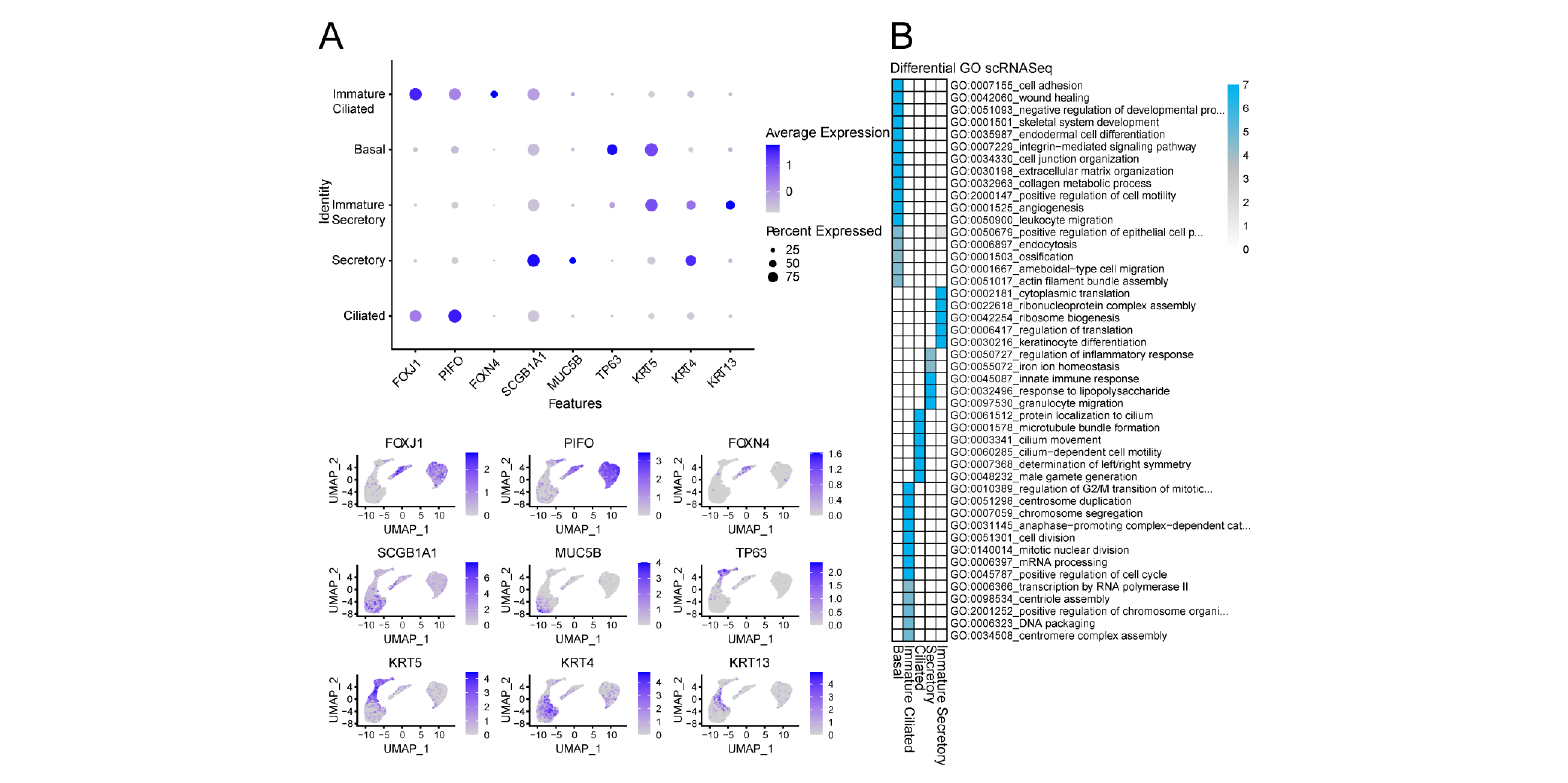

Supplement: S1 Fig — (A) The expression of marker genes of lung epithelial cell types is shown for each cluster defined from the scRNA–seq reads of AirGels. Average expression per cluster and the percentage of cells expressing the respective marker gene. (B) Subset–specific expression of canonical marker genes on UMAP embedding. FOXJ1 and PIFO are typically expressed in ciliated cells. The immature ciliated cell cluster, also known as deuterosomal cells, is marked by high levels of FOXJ1 and expression of FOXN4. Basal cells typically express TP63 and KRT5. The secretory cluster shows expression of SCGB1A1 and a fraction of more mature secretory cells expressing MUC5B [89]. Furthermore, we observe a transitional state between basal and secretory, the immature secretory cluster, which shows partial mutual expression of KRT4 and KRT13 as previously described [40]. (C) A gene ontology (GO) analysis was performed on the most differentially expressed genes in each cluster confirming the correct annotation of cell clusters. (PNG) [file pbio.3002209.s001.png]

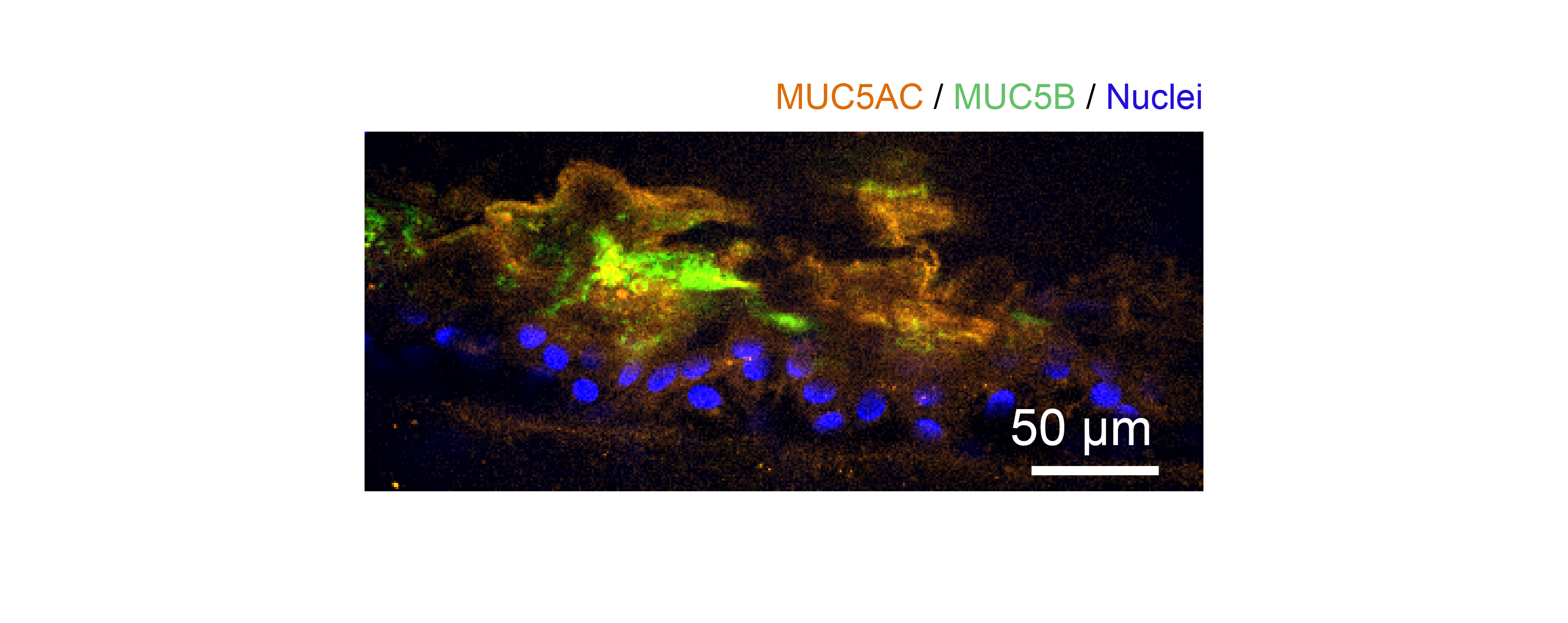

Supplement: S2 Fig — Luminal mucus in a methacarn–fixed AirGel. Staining was done with antibodies against MUC5AC and MUC5B gel–forming mucins. (PNG) [file pbio.3002209.s002.png]

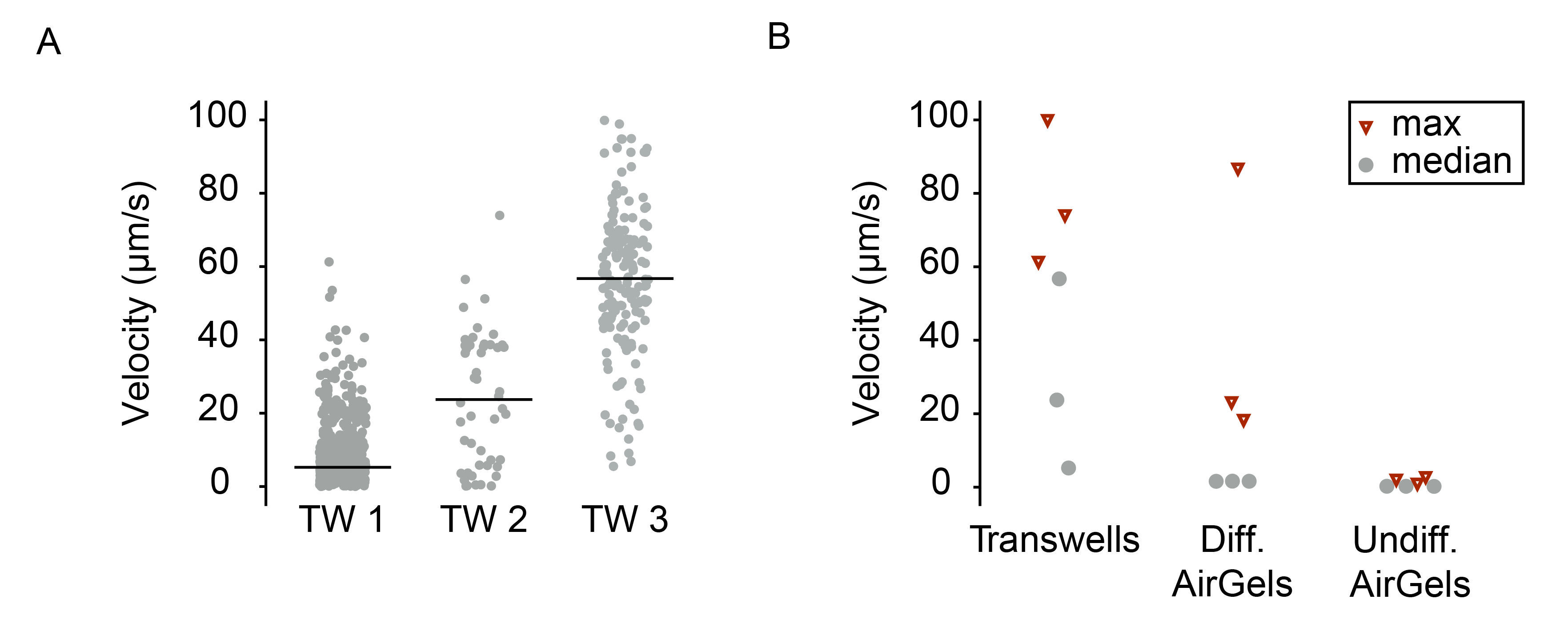

Supplement: S3 Fig — (A) Velocities of fluorescent microparticles transported by the cilia of healthy airway epithelia growing on Transwell (TW) membranes. (B) Maximal (red triangles) and median (gray circles) velocities of fluorescent microparticles for N = 3 Transwells, differentiated AirGels, and undifferentiated AirGels, respectively. The low median values for differentiated AirGels can be at least partly explained by the curvature of the lumen: when we image AirGels with a confocal microscope, we simultaneously visualize particles at varying distances of the epithelium. Only a fraction of these particles lies within the distance that allows for maximal clearance, thereby biasing population–level velocities to lower values. (PNG) [file pbio.3002209.s003.png]

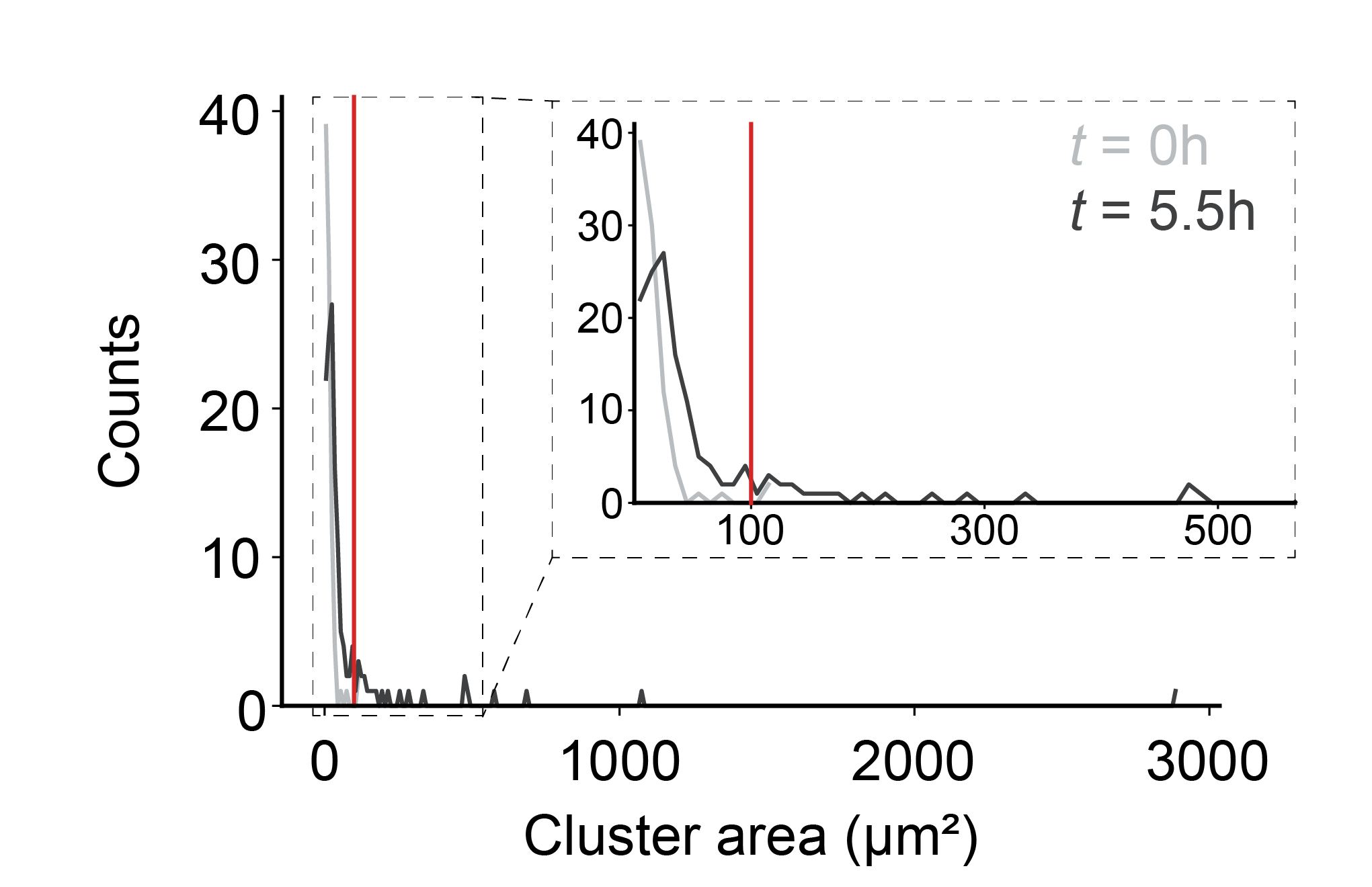

Supplement: S4 Fig — The red line indicates the threshold for what we considered as large clusters (>100 μm2). (PNG) [file pbio.3002209.s004.png]

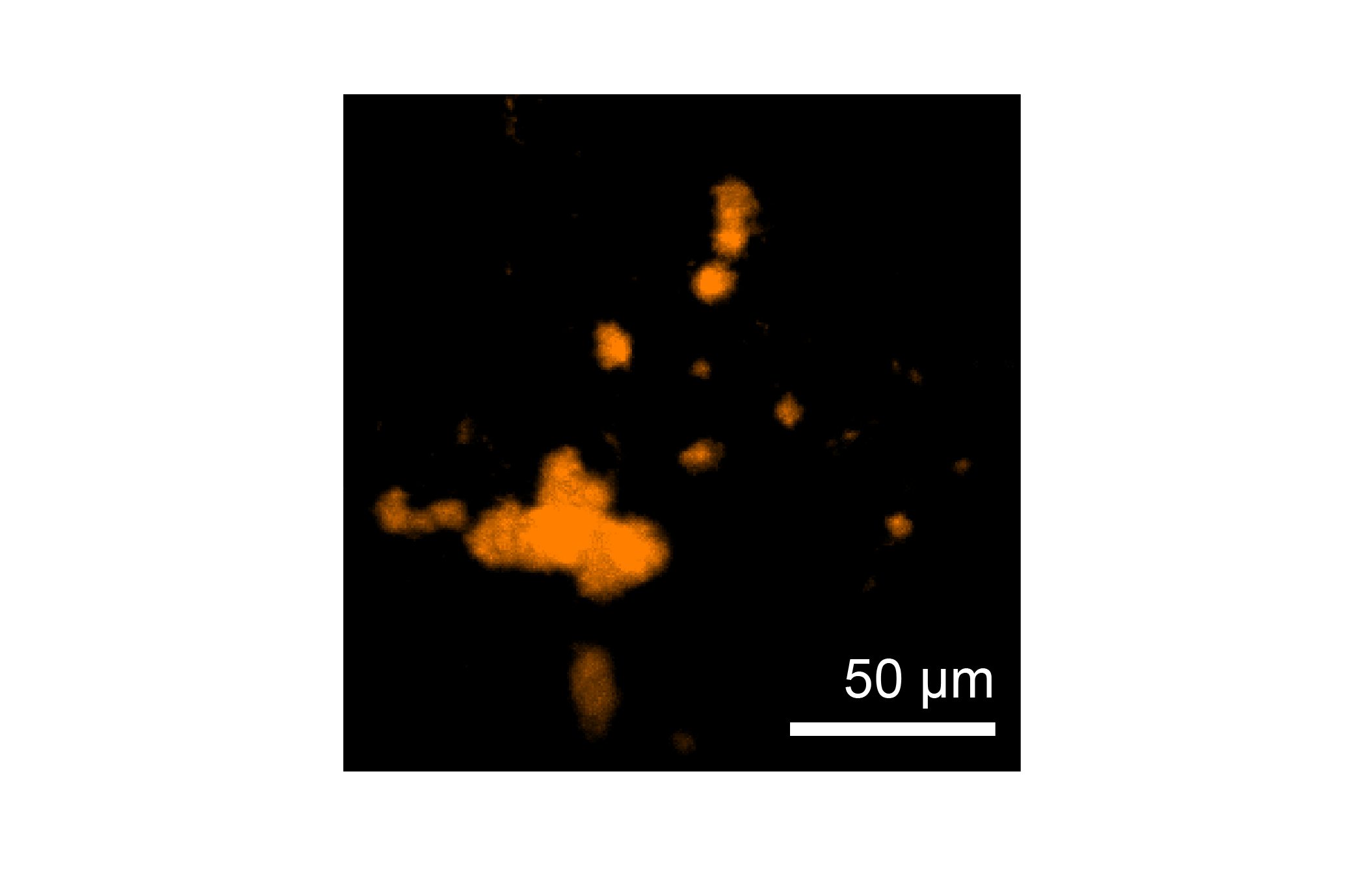

Supplement: S5 Fig — After 4 h 15, bacterial aggregates were already visible, indicating their formation is independent of jacalin staining. (PNG) [file pbio.3002209.s005.png]

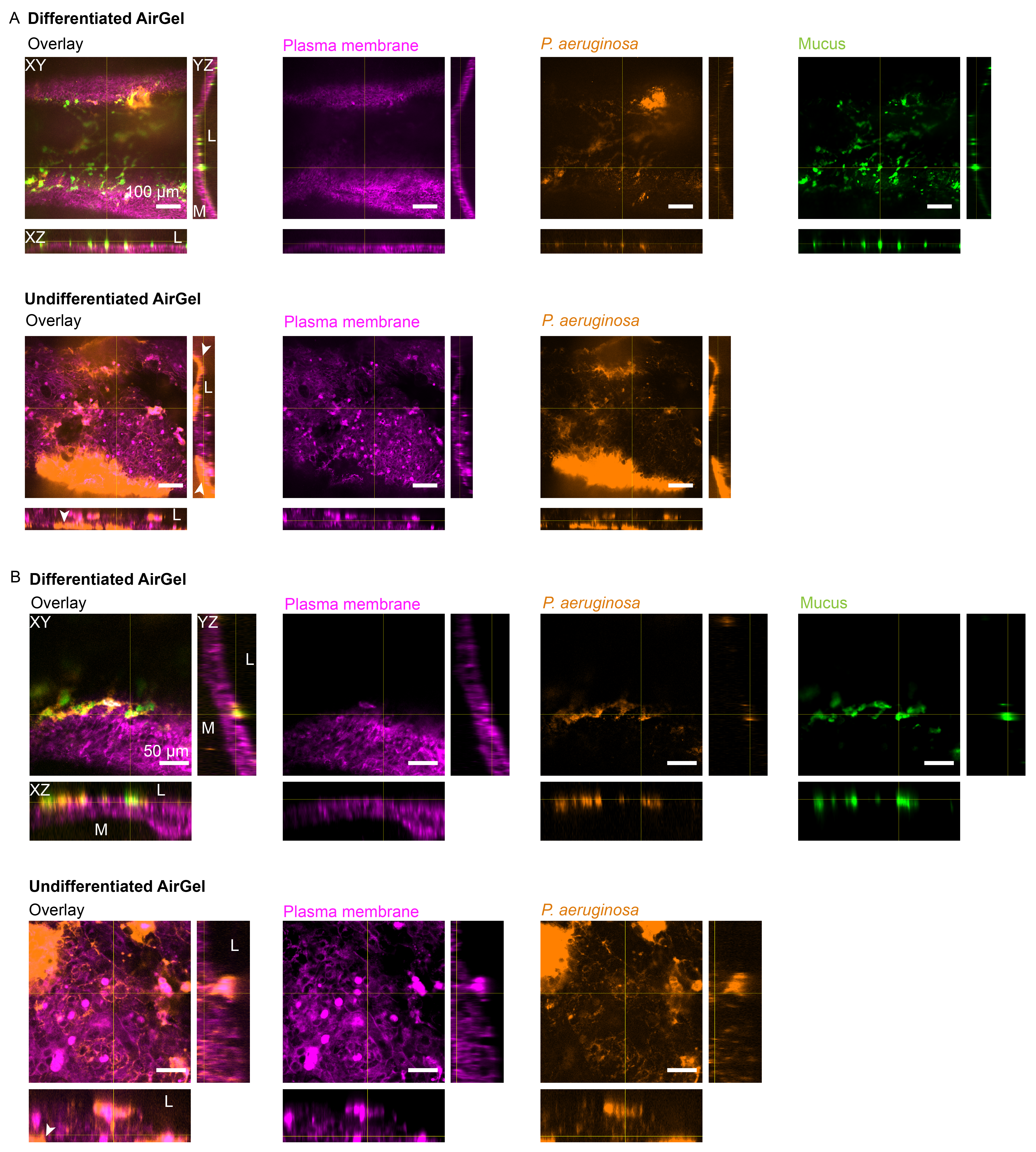

Supplement: S6 Fig — Orthogonal views of infections with P. aeruginosa in a differentiated versus an undifferentiated AirGel stained with Jacalin at low (A) and high (B) magnification. The infection was imaged at t = 5.5 h (differentiated) and t = 6 h (undifferentiated) post–inoculation. L indicates the luminal side and M the extracellular matrix. We did not observe P. aeruginosa aggregates on the luminal side of the undifferentiated AirGel. However, bacteria damaged the epithelium extensively in the absence of mucus, which resulted in invasion of the extracellular matrix (white arrowheads). Magenta: plasma membrane; green: mucus; orange: P. aeruginosa. (PNG) [file pbio.3002209.s006.png]

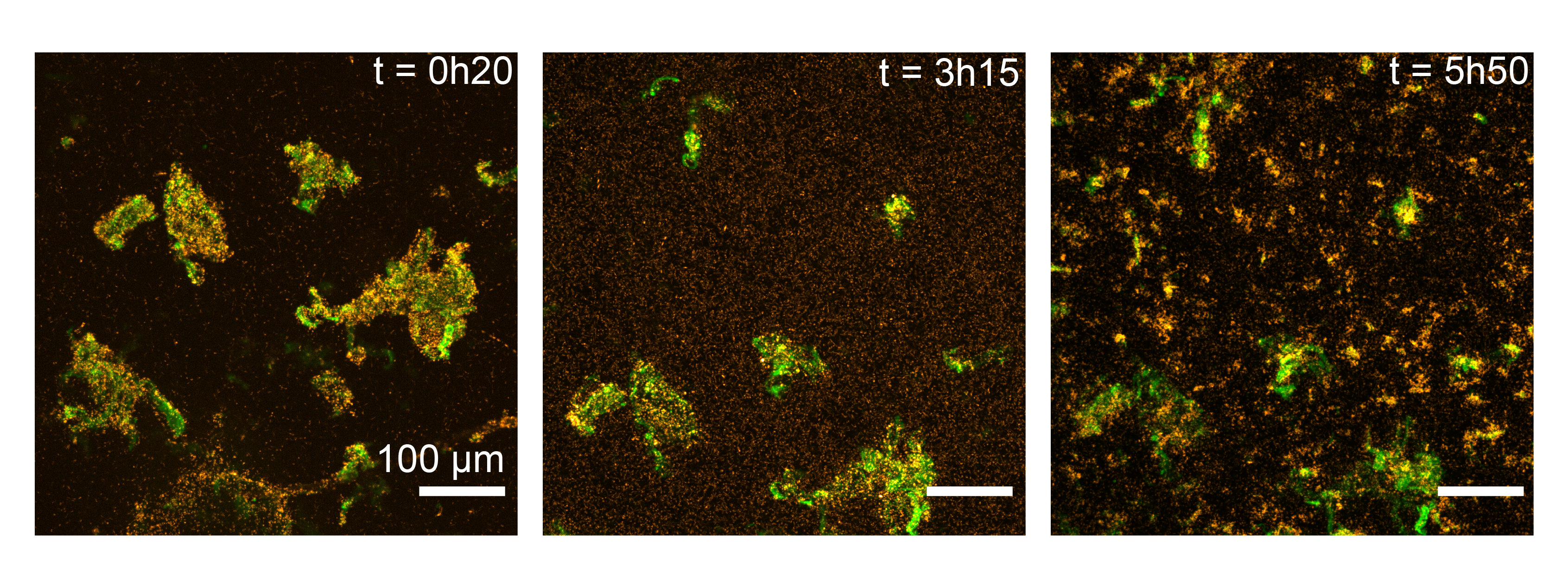

Supplement: S7 Fig — Jacalin–labeled mucus that had been isolated from a differentiated HBE culture on a Transwell. Even after almost 6 h after incubation with P. aeruginosa, no aggregates were visible. (PNG) [file pbio.3002209.s007.png]

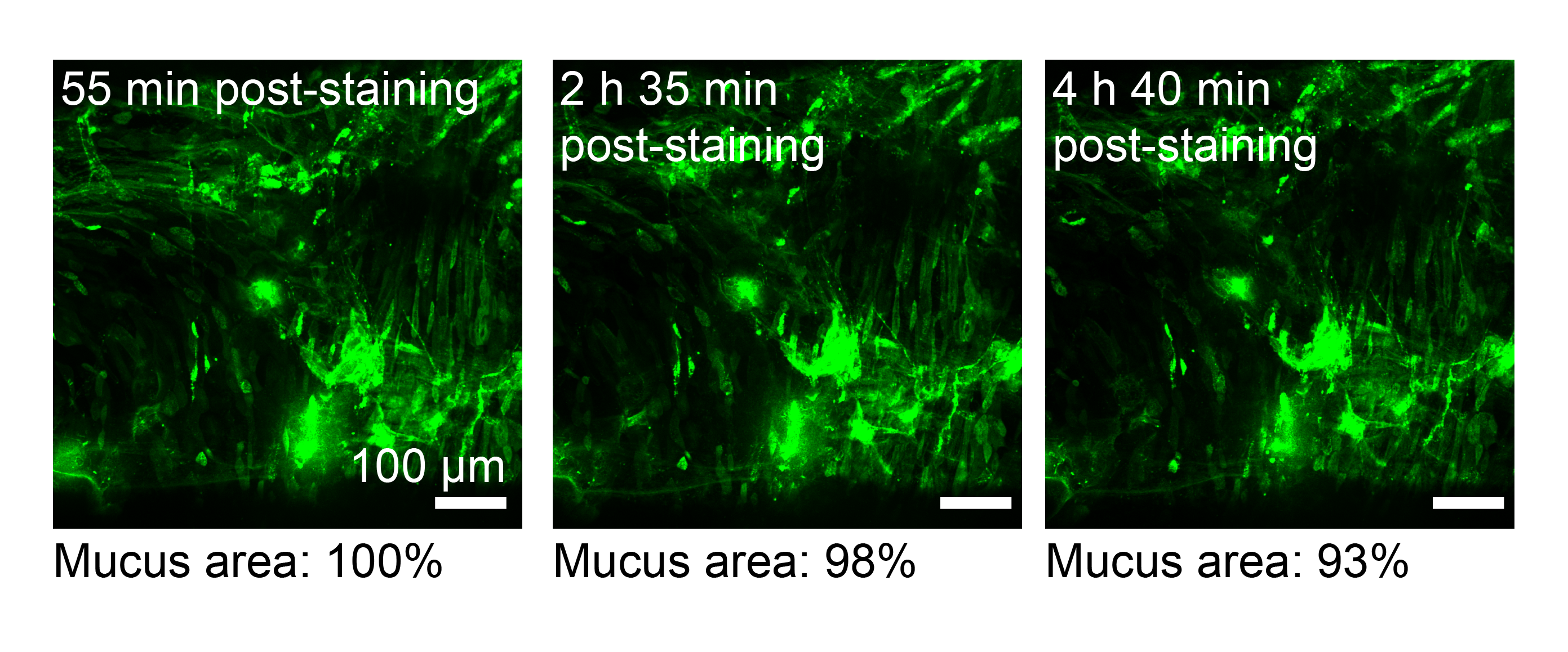

Supplement: S8 Fig — Jacalin–stained mucus in an uninfected AirGel. The total area of mucus was estimated over time and found to only differ slightly over time, most likely due to photobleaching and drift in and out of focus. (PNG) [file pbio.3002209.s008.png]

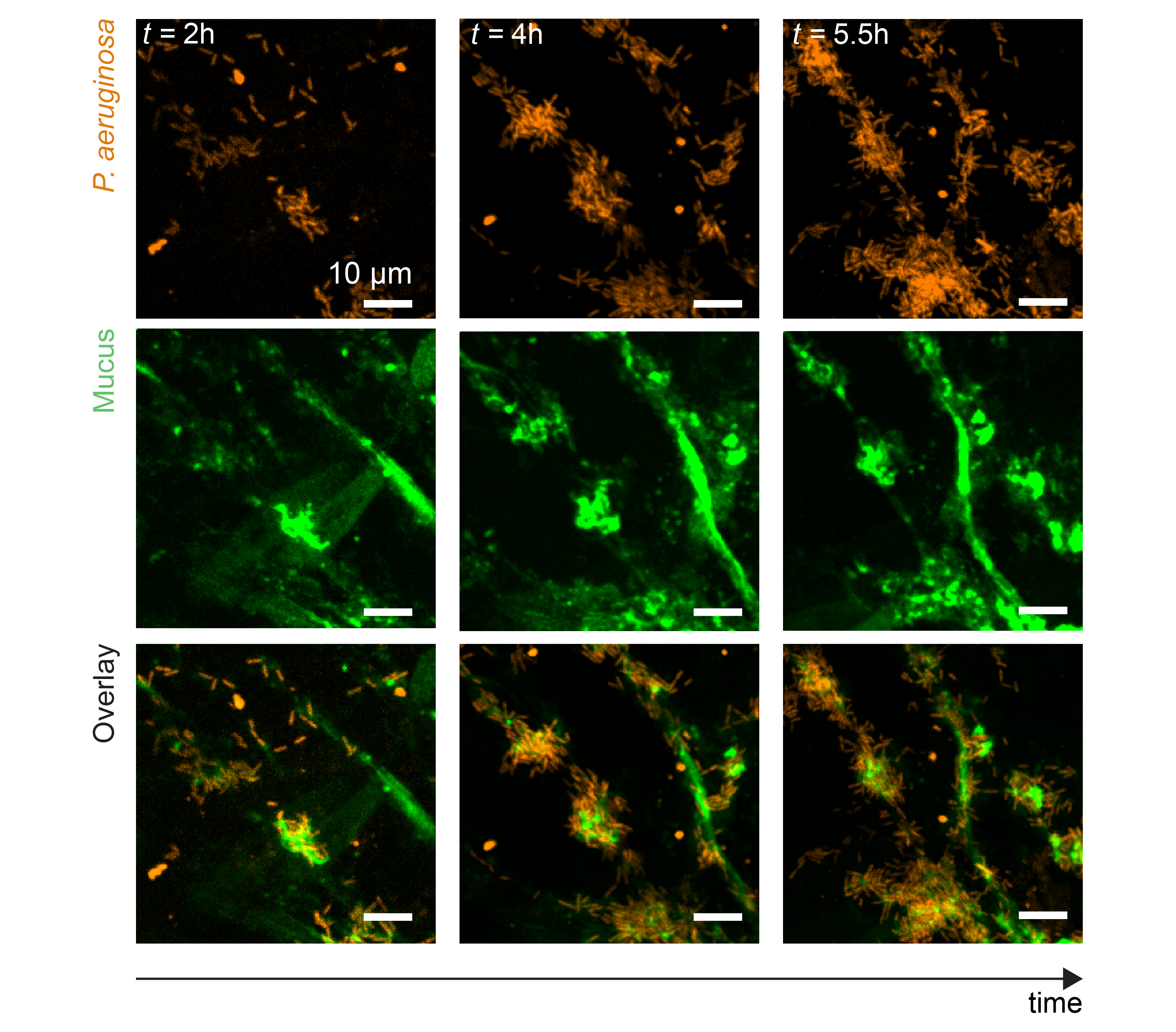

Supplement: S9 Fig — This strain came from the bronchoalveolar lavage fluid of a cystic fibrosis patient. Micrographs show a time course during which bacteria (orange) contract mucus (green). All images are maximal intensity projections from z–stacks. (PNG) [file pbio.3002209.s009.png]

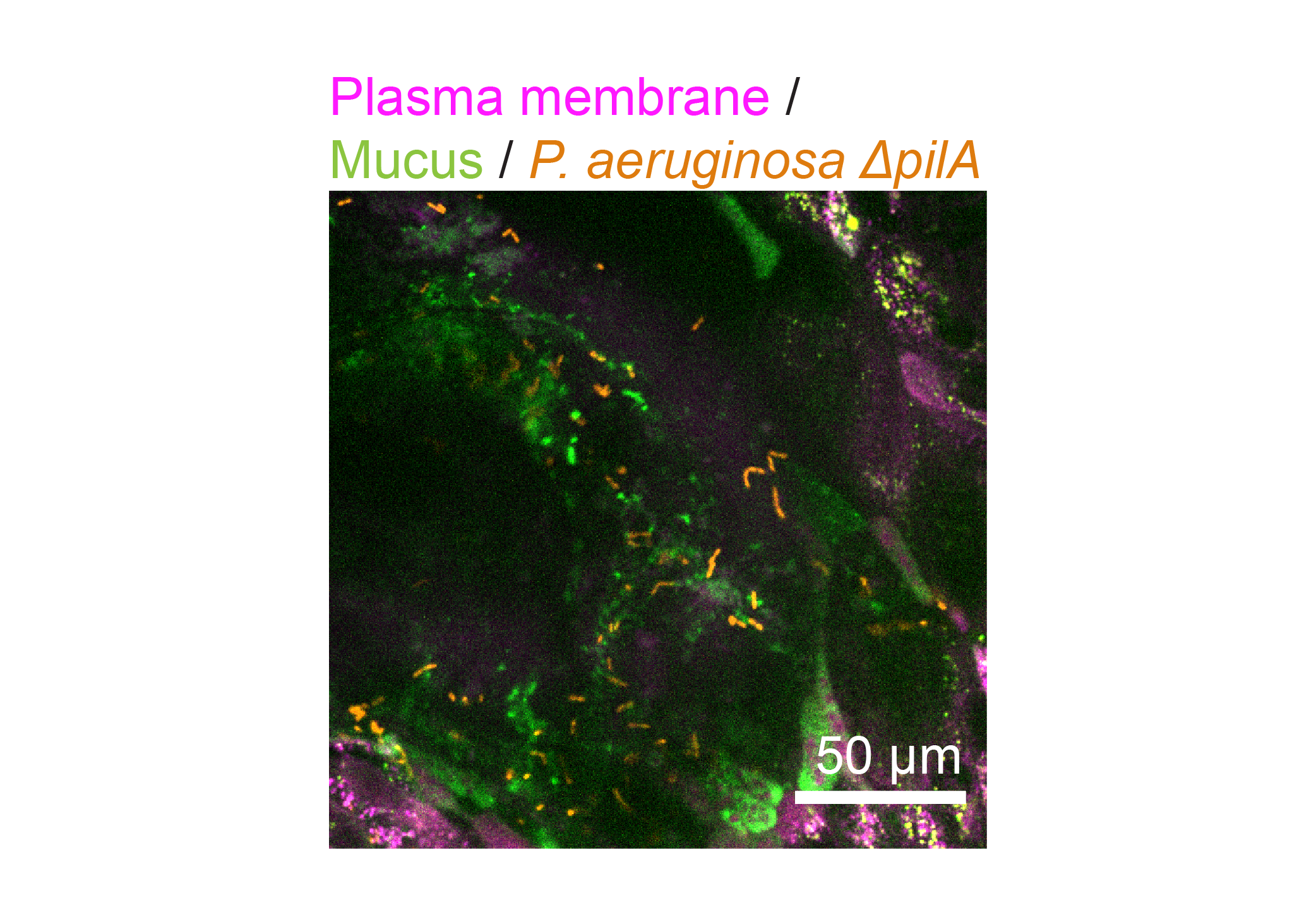

Supplement: S10 Fig — Aggregates of the ΔpilA mutant P. aeruginosa were still absent 3 h 25 post–inoculation. However, the bacteria colocalized with the mucus, indicating that T4P are not necessary for adhesion to mucus. (PNG) [file pbio.3002209.s010.png]

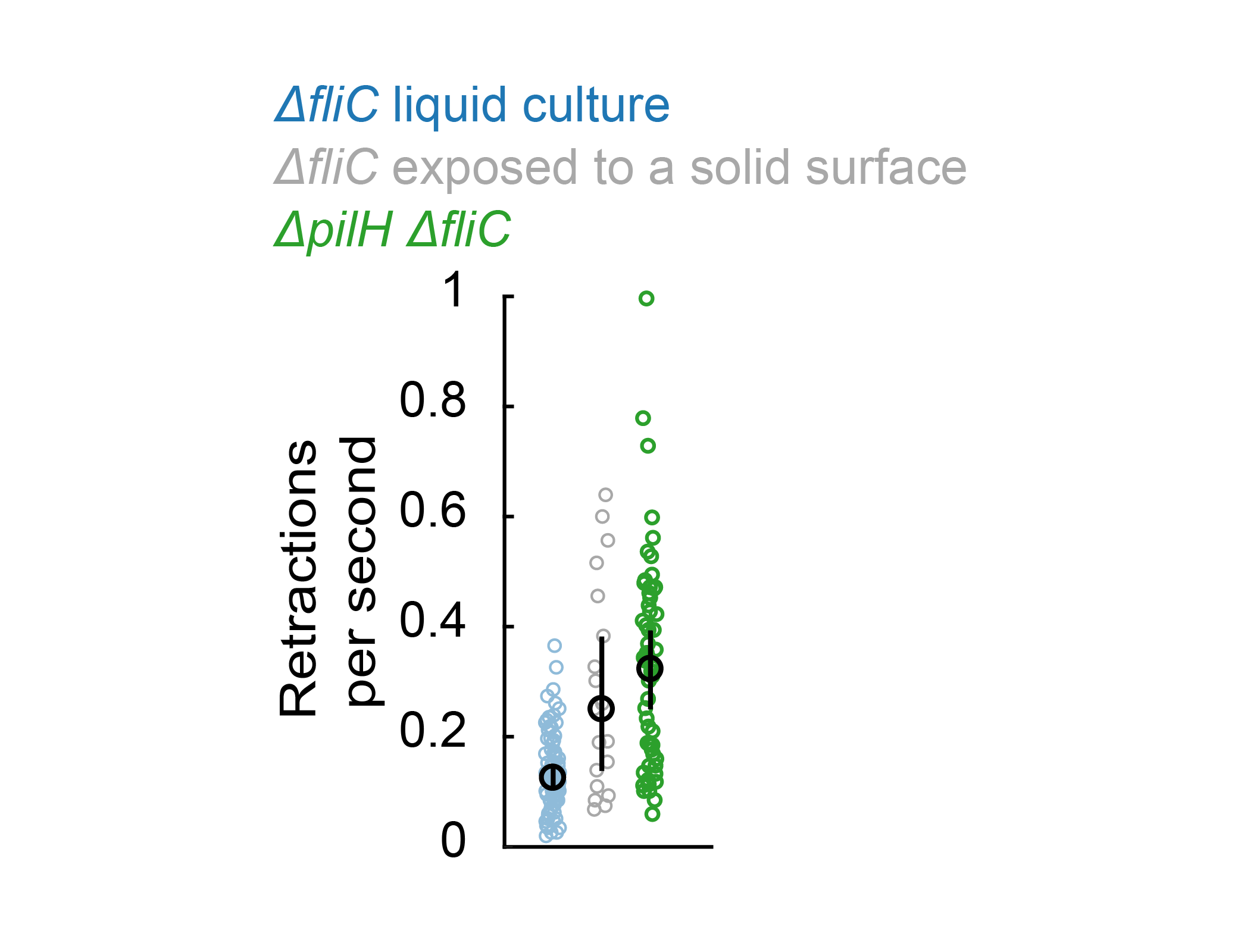

Supplement: S11 Fig — T4P retraction rates were measured by interferometric scattering (iSCAT) microscopy, which allows for label–free T4P visualization [59]. To prevent cells from swimming away during the iSCAT measurements, a flagellum–less ΔfliC mutant was used as background strain. This strain was either grown in liquid or preadapted to culture on a solid surface for 3 h. Black circles and bars indicate the bootstrap median and 95% confidence interval of the medians, respectively. (PNG) [file pbio.3002209.s011.png]
