# Supplementary material for: Pseudomonas aeruginosa type IV pili actively induce mucus contraction to form biofilms in tissue-engineered human airways
Source: PLoS Biol. 2023 Aug 1;21(8):e3002209. doi: 10.1371/journal.pbio.3002209 (PMC10393179; doi:10.1371/journal.pbio.3002209)
Supplement: S1 Table — (DOCX) [file pbio.3002209.s032.docx]

**S1 Table: strains used in this study**

| **Strain** | **Relevant characteristics** | **Source / Reference** |
| --- | --- | --- |
| *Pseudomonas aeruginosa* PAO1 (ATCC 15692) | WT PAO1 | ^1^ |
| PAO1 mScarlet | WT PAO1 with constitutive chromosomal mScarlet expression | This study |
| PAO1 *Δxcp* | *xcpP* to *xcpZ* chromosomal deletion (called DZQ40 in the original study) | ^2^ |
| PAO1 *Δxcp* mScarlet | PAO1 *Δxcp* with constitutive chromosomal mScarlet expression | This study |
| PAO1 *ΔfliC* | In-frame deletion of PA1092 | ^3^ |
| PAO1 *ΔfliC* mScarlet | PAO1 *ΔfliC* with constitutive chromosomal mScarlet expression | This study |
| PAO1 *ΔpilA* | In-frame deletion of *pilA* | ^4^ |
| PAO1 *ΔpilA* mScarlet | PAO1 *ΔpilA* with constitutive chromosomal mScarlet expression | This study |
| PAO1 *ΔpilT* | *pilT*::Tn5 | ^5^ |
| PAO1 *ΔpilT* mScarlet | PAO1 *ΔpilT* with constitutive chromosomal mScarlet expression | This study |
| PAO1 *ΔpilH* | In-frame deletion of PA0409 | ^6^ |
| PAO1 *ΔpilH* mScarlet | PAO1 *ΔpilH* with constitutive chromosomal mScarlet expression | This study |
| PAO1 *ΔfliC ΔpilH* | In-frame deletion of PA1092 and PA0409 | ^7^ |
| *P. aeruginosa* AP1889 | Strain isolated from the bronchoalveolar lavage fluid of a cystic fibrosis patient | Shared by Prof. Dr. Dr. Adrian Egli, Institute of Medical Microbiology, University of Zurich, Switzerland |
| *P. aeruginosa* AP1913 | AP1889 isolate with constitutive chromosomal mScarlet expression | This study |

**References**

1. Holloway, B. W. & Morgan, A. F. Genome Organization in Pseudomonas. *Annual Review of Microbiology* **40**, 79–105 (1986).

2. Ball, G., Chapon-Hervé, V., Bleves, S., Michel, G. & Bally, M. Assembly of XcpR in the Cytoplasmic Membrane Is Required for Extracellular Protein Secretion in Pseudomonas aeruginosa. *Journal of Bacteriology* **181**, 382–388 (1999).

3. Bertrand, J. J., West, J. T. & Engel, J. N. Genetic Analysis of the Regulation of Type IV Pilus Function by the Chp Chemosensory System of Pseudomonas aeruginosa. *Journal of Bacteriology* **192**, 994–1010 (2010).

4. Cowles, K. N. *et al.* The putative Poc complex controls two distinct Pseudomonas aeruginosa polar motility mechanisms. *Molecular Microbiology* **90**, 923–938 (2013).

5. Jacobs, M. A. *et al.* Comprehensive transposon mutant library of Pseudomonas aeruginosa. *Proc Natl Acad Sci U S A* **100**, 14339–14344 (2003).

6. Barken, K. B. *et al.* Roles of type IV pili, flagellum-mediated motility and extracellular DNA in the formation of mature multicellular structures in Pseudomonas aeruginosa biofilms. *Environmental Microbiology* **10**, 2331–2343 (2008).

7. Kühn, M. J. *et al.* Mechanotaxis directs Pseudomonas aeruginosa twitching motility. *Proceedings of the National Academy of Sciences* **118**, e2101759118 (2021).
