# Supplementary material for: Pseudomonas aeruginosa type IV pili actively induce mucus contraction to form biofilms in tissue-engineered human airways
Source: PLoS Biol. 2023 Aug 1;21(8):e3002209. doi: 10.1371/journal.pbio.3002209 (PMC10393179; doi:10.1371/journal.pbio.3002209)
Supplement: S2 Table — (DOCX) [file pbio.3002209.s033.docx]

**S2 Table: plasmids used in this study**

| **Plasmid** | **Source** | **Reference** |
| --- | --- | --- |
| pTNS2 | Addgene 64968 | ^1^ |
| pUC18T-mini-Tn7T-Gm- Ptet_mScarlet | Addgene 63121 with Ptet promoter fused to mScarlet | This study |

**References**

1. Choi, K.-H. *et al.* A Tn7-based broad-range bacterial cloning and expression system. *Nat Methods* **2**, 443–448 (2005).
