# Supplementary material for: Pseudomonas aeruginosa type IV pili actively induce mucus contraction to form biofilms in tissue-engineered human airways
Source: PLoS Biol. 2023 Aug 1;21(8):e3002209. doi: 10.1371/journal.pbio.3002209 (PMC10393179; doi:10.1371/journal.pbio.3002209)
Supplement: S3 Table — (DOCX) [file pbio.3002209.s034.docx]

**S3 Table: growth of different *P. aeruginosa* mutants on airway mucus**

The differences in generation numbers were not statistically significant (one-way ANOVA, *p* = 0.46).

| **Strain** | **Mean number of generations ± standard deviation** | **Growth rate (generations · h^-1^)** |
| --- | --- | --- |
| WT | 4.64 ± 0.38 | 0.928 ± 0.076 |
| *ΔpilT* | 4.34 ± 0.61 | 0.868 ± 0.122 |
| *ΔpilH* | 4.08 ± 0.16 | 0.816 ± 0.032 |
