# Supplementary material for: Pseudomonas aeruginosa type IV pili actively induce mucus contraction to form biofilms in tissue-engineered human airways
Source: PLoS Biol. 2023 Aug 1;21(8):e3002209. doi: 10.1371/journal.pbio.3002209 (PMC10393179; doi:10.1371/journal.pbio.3002209)
Supplement: Attachment — Submitted filename: Response to reviewers.docx [file pbio.3002209.s035.docx]

Response to reviewers’ comments for:

*Pseudomonas aeruginosa* contracts mucus to rapidly form biofilms in tissue-engineered human airways

**Authors:**

Tamara Rossy^1,2^, Tania Distler^1,2^, Lucas A. Meirelles^1,2^, Joern Pezoldt^2^, Jaemin Kim^3^, Lorenzo Talà^1,2^, Nikolaos Bouklas^3^, Bart Deplancke^2^, Alexandre Persat^1,2^

**Affiliations:**

^1^ Global Health Institute, School of Life Sciences, Ecole Polytechnique Fédérale de Lausanne (EPFL), Lausanne, Switzerland

^2^ Institute of Bioengineering, School of Life Sciences, École Polytechnique Fédérale de Lausanne (EPFL), Lausanne, Switzerland

^3^ Sibley School of Mechanical and Aerospace Engineering, Cornell University, Ithaca, NY, USA

**Correspondence:** [alexandre.persat@epfl.ch](mailto:alexandre.persat@epfl.ch)

# Editor’s comments:

In light of the reviews, which you will find at the end of this email, we would like to invite you to revise the work to thoroughly address the reviewers' reports.

As you will see below, the reviewers find the manuscript interesting but raise some issues that should be solved before publication. Please address all the reviewers' issues including the control experiments with the natural isolate of P. aeruginosa from the CF patient, as requested by the reviewer #2.

Given the extent of revision needed, we cannot make a decision about publication until we have seen the revised manuscript and your response to the reviewers' comments. Your revised manuscript is likely to be sent for further evaluation by all or a subset of the reviewers.

# Reviewer #1’s comments:

Rossy et al establish a novel lung model system for studying Pseudomonas aeruginosa infection. This model system enables 3D live imaging of bacteria. The authors confirm that after differentiation, epithelia are protected from damage caused by P. aeruginosa as expected for mucus producing epithelia. They show that within this model system, P. aeruginosa forms biofilms at a time scale of hours, mostly driven by fusion of colonies. Importantly, the time scale of biofilm formation is much faster than has been observed in vitro so far. The authors identify type IV pilus activity as the mechanism for mucus contraction and colony fusion. They conclude that mucus formation comes with a trade-off; while it protects the epithelia from bacterial invasion, it facilitates biofilm formation.

I am no expert on infection models, but the model developed in this study looks intriguing, since it reveals bacterial behavior that is not reproducible in vitro. Overall, I believe the authors established a highly promising infection model that will serve the community to address follow up questions about biofilm formation by an important model organism. The result that type IV activity is required for rapid biofilm formation in the close to natural infection environment are both convincing and interesting. I have only a few minor issues that hopefully help improving the manuscript.

*We thank the reviewer for the positive comments on our manuscript.*

1. While the model shows previously unseen behavior, it remains unclear whether mucus contraction affects pathogenicity of P. aeruginosa. It would be good to discuss this point.

*We agree with the reviewer and added a discussion of this point at lines 266-277. We particularly discuss the potential for contraction-induced biofilm formation in reducing the proportion of planktonic P. aeruginosa which mostly contribute to cytotoxicity.*

2. Line 230: "After this initial biofilm seeding, *P. aeruginosa* may initiate the secretion of matrix components to strengthen the cohesion of the biofilm." On which experimental hint or evidence is this speculation based? Since this speculation is not required for further discussion I suggest omitting it.

*We understand the reviewer’s point. We removed that line from the discussion.*

3. The dynamics of colony formation and fusion are astonishingly similar to colony formation of Neisseria species in vitro even in the absence of mucus. It would be interesting to discuss this analogy. (doi.org/10.1016/j.cell.2018.04.010 and doi.org/10.1103/PhysRevLett.121.118102)

*We thank the reviewer for this comment. Indeed, there are similarities in the sense that in both systems, the emergence of contractile forces from T4P improve the cohesion between cells. We now discuss this point at lines 285-290.*

4. There is no reference to Supplementary Fig. 2a. Why is its shown? Is it part of the control for jacalin labeling?

*We realized that showing these two panels in the same figure was confusing, since they are not directly related. Therefore, we split them into two (Supplementary Figures 4 & 5). We also want to point out that we did refer to Supplementary Figure 4 (formerly 2a) in the text (methods section, “Biofilm image acquisition and analysis”, p.18). However, we also decided to refer to it in the main text, line 140.*

5. Supplementary Fig. 3: It would be good to show the fluorescence signals of bacteria (orange) and membrane? (pink) in different panels. It is difficult to distinguish these colors. Also, please explain what the colors refer to the Figure legend.

*We applied the changes required by the reviewer. The resulting figure is now called Supplementary Figure 6.*

6. Supplementary Fig. 8: I understand that the total fluorescence intensity is used as a measure for total cell number. If this is correct then the there seems to be no net growth. How can you claim that the growth rate is not influenced by mutations? What means N = 9? There are only three curves per strain. Please correct to N = 3 if there are only three curves per strain.

*We thank the reviewer for that observation. We realized that image-based quantification of biomass was misleading, because we observed consistent cell divisions despite what our graph suggested. We attribute this error to drift in the z direction and photobleaching effects. To overcome these limitations, we replaced this figure with a more precise quantification of bacterial growth at the airway mucosal surface using colony-forming units, which rigorously demonstrate that these strains grow. The results and corresponding methods can be found on p. 17 of the supplementary materials (Supplementary Table 3 and paragraph “Quantification of bacterial growth on airway mucus”). In addition, they are referred to in the main text, lines 229-232.*

# Reviewer #2’s comments:

This paper connects important airway biology and microbiology processes in a way that is novel, fills a gap in the field, and addresses previously unknown mechanisms of bacterial function and disease. There are, however, some gaps in the description of the methods and in the results that should be addressed before publication.

1. Figure 1 presents an interesting and innovative method of culturing airway epithelial cells in a more physiologically relevant way. However, it's not clear how the cells are able to maintain air-liquid interface. The diagram in Figure 1b shows a luminal access port, but how is this port protected from the surrounding media and how is air moved in? Is there a pressure pump moving air across? Perhaps a picture of the actual device rather than a graphic would make the ALI more clear.

*The inlet port is physically separated from the basal medium by PDMS. The air-liquid interface is generated by aspirating the medium out of the lumen, and then maintained passively, i.e. without the need for pumping. To make it clearer, we replaced the top view schematic in Figure 1b with a picture of an AirGel chip, and added a sentence on line 93. We refer the reviewer to p.12 of the supplementary material (“Long-term culture in AirGels”) for more details.*

2. The methods section indicates that HBEs were obtained from both CF and non-CF sources. Which were used in the data presented here?

*As indicated on p. 15 of the supplementary material, “Aside from the infection assay shown in Figure 3a and Supplementary Video 2, which was performed in a CF AirGels, all infections were run with cells from healthy donors.”*

3. Figure 2 nicely shows appropriate architecture. However, MUC5AC and MUC5B should be differentially stained, to understand which mucin is primarily expressed. This is particularly important for the later figures, because previous studies have shown that the arrangement of mucins in the airway strands may influence disease.

*We thank the reviewer for this suggestion. We performed the suggested experiments with a new antibody for MUC5AC to perform simultaneous visualization with MUC5B. The result is shown in Supplementary Figure 2, which highlights the presence of the two mucins in mucus. There is no obvious difference in mucin levels based on immunofluorescence, but we did notice they have a distinct spatial structure.*

4. In figure 2 iii, the measured mucociliary clearance is very slow. This is important because the rate of mucus clearance could affect biofilm formation. The references cited to defend this rate as physiologic are a very small subset of many studies measuring MCC ex vivo, (one of the papers cited is a review, which does not present primary data) and one of the papers present MCC data that is ten-fold higher than the data presented here. The authors should include more references to capture a more complete picture of the data found in the field, and a comparison of MCC and ciliary beat frequency measured by these methods in flat monolayers, which have been shown to closely represent ciliary function, would be needed to ensure that the method of culturing is not affecting mucus and cilia movement. This is particularly important given that these host factors may influence mucus contraction in addition to the bacterially-driven mechanisms proposed later in the study.

*Following the reviewer’s suggestion, we repeated the measurements of MCC in Transwells to compare them with the values obtained in AirGels. The results are shown in Supplementary Figure 3. In addition, we incorporated a more nuanced discussion on lines 116-123.*

*To summarize, the MCC values in AirGels are indeed lower than in Transwells, but we attribute that to:*

*1) the curvature of AirGels, which results in heterogeneous velocities (in one confocal slice, we simultaneously image microparticles bound to cilia, others moving with the mucus, and others floating far above the epithelium). The median values are thus an underestimate of the actual clearance velocity. We want to point out that maximal velocities (Supplementary Figure 3b) are comparable between Transwells and AirGels.*

*2) low ambient humidity in the lumen of AirGels.*

5. There is some important information missing from the methods regarding the infections. At what CFU are the bacteria inoculated? And at what stage of growth? The formation of biofilms in the mucus may be dependent on if the bacteria are collected in the doubling or stationary phases.

*We inoculated approximately 10^6^ bacteria for our infection experiments. In addition, our method section states “We measured the optical density of our* ***exponential*** *bacterial cultures and centrifuged them for 2-3 min at 5000 rpm. We discarded the supernatant and resuspended the pellet in D-PBS to reach an optical density value of approximately 3. We then loaded 0.5 μl of bacterial culture in the lumen of AirGels (this small volume allowed for ALI maintenance). The resulting multiplicity of infection was approximately 10 (~10^6^ bacteria for ~10^5^ airway cells).” To clarify this point, we added some of the details to the main text (line 137).*

6. The study as presented is convincing, particularly around with the mutants. However, confirmatory studies showing this relationship with mucus in a non-PAO1 strain would be a critical component. Of particular interest would be a lung-adapted strain. This is vital, because patients, especially with CF and other muco-obstructive disorders, have cultured isolates of P. aeruginosa that are phenotypically and mechanistically very different from PAO1.

*We thank the reviewer for this suggestion. We obtained a clinical strain isolated from the bronchoalveolar lavage of a cystic fibrosis patient (gift from the Egli lab, Zurich), and used it to infect an AirGel. We observed a similar behavior as in PAO1, with rapid colonization and contraction of mucus (Supplementary Figure 9, Supplementary video 6). The corresponding description in the main text is on lines 178-182.*
