# Supplementary material for: Pseudomonas aeruginosa type IV pili actively induce mucus contraction to form biofilms in tissue-engineered human airways
Source: PLoS Biol. 2023 Aug 1;21(8):e3002209. doi: 10.1371/journal.pbio.3002209 (PMC10393179; doi:10.1371/journal.pbio.3002209)
Supplement: Attachment — Submitted filename: response_to_reviewers_2.docx [file pbio.3002209.s036.docx]

**Requests from 06.06.2023:**

Based on our Academic Editor's assessment of your revision, we are likely to accept this manuscript for publication, provided you satisfactorily address the following data and other policy-related requests.

a) The unusual transitive use of "contracts" in the Title may cause confusion. Please change your Title to "Pseudomonas aeruginosa type IV pili actively induce mucus contraction to form biofilms in tissue-engineered human airways"

*We changed the title according to the editor’s suggestion.*

b) Please address my Data Policy requests below; specifically, we need you to supply the numerical values underlying Figs 2AB, 3, 4BC, S1, S2, S3, S5ABCD, S6AB, either as a supplementary data file or as a permanent DOI’d deposition.

*We now provide all the raw data as supplementary material.*

c) Please cite the location of the data clearly in all relevant main and supplementary Figure legends, e.g. “The data underlying this Figure can be found in S1 Data” or “The data underlying this Figure can be found in <https://doi.org/10.5281/zenodo.XXXXX>”

*We now cite the location of the raw data in the legends.*

d) We note that your Materials and Methods section is currently in the Supplement – please move it to the main manuscript.

*We moved the Materials and Methods to the main text.*

**Requests from 19.06.2023:**

1.Please ensure that the credits / author contributions listed for every co-author are completed accurately and in full. At this stage all authors require listed contributions. Please can you confirm the contributions via email and we would be happy to add them on your behalf. If any authors do not meet any criteria for authorship please let us know as soon as possible. CRediT author contributions are: Conceptualization, Data curation, Formal analysis, Funding acquisition, Investigation, Methodology, Project administration, Resources, Software, Supervision, Validation, Visualization, Writing – original draft, Writing – review & editing.

*We have verified that the contributions of all the authors are listed accurately in the submission portal.*

2.Please upload the latest version of your ‘Response to Reviewers’ file. We have noticed that your latest ‘Response to Reviewers’ file was uploaded on May 30, whereas your latest Manuscript file was uploaded on June 17.

*We now provide the current file.*

3. Some material included in your submission may be copyrighted. According to PLOS’s copyright policy, authors who use figures or other material (e.g., graphics, clipart, maps) from another author or copyright holder must demonstrate or obtain permission to publish this material under the Creative Commons Attribution 4.0 International (CC BY 4.0) License used by PLOS journals. Please closely review the details of PLOS’s copyright requirements here: PLOS Licenses and Copyright. If you need to request permissions from a copyright holder, you may use PLOS's Copyright Content Permission form.

Please respond directly to this email and provide any known details concerning your material's license terms and permissions required for reuse, even if you have not yet obtained copyright permissions or are unsure of your material's copyright compatibility. Once you have responded and addressed all other outstanding technical requirements, you may resubmit your manuscript within Editorial Manager.

Potential Copyright Issues:

-“ Figure 1B”: Please confirm (a) that you are the photographer; or (b) provide written permission from the photographer to publish the photo(s) under our CC BY 4.0 license.

*The authors are the photographers who took Figure 1b.*

- “Figure 1A” and “Figure 5B”: Please confirm whether you drew the images / clip-art within the figure panels by hand. If you did not draw the images, please provide (a) a link to the source of the images or icons and their license / terms of use; or (b) written permission from the copyright holder to publish the images or icons under our CC BY 4.0 license. Alternatively, you may replace the images with open source alternatives.

*The authors drew Figure 1a and Figure 5b manually.*
